# Evaluation of a set of refolded recombinant antigens for serodiagnosis of human fascioliasis

**DOI:** 10.1371/journal.pone.0203490

**Published:** 2018-10-03

**Authors:** Abolfazl Mirzadeh, Asiyeh Yoosefy, Elham Kazemirad, Zahra Barati, Majid Golkar, Jalal Babaie, Farid Jafarihaghighi, Zarrintaj Valadkhani

**Affiliations:** 1 Department of Medical Parasitology, Pasteur Institute of Iran, Tehran, Iran; 2 Department of Medical Parasitology and Mycology, School of Public Health, Tehran University of Medical Sciences, Tehran, Iran; La Trobe University, AUSTRALIA

## Abstract

Diagnosis of fascioliasis with high sensitivity and specificity antigens play a vital role in the management of the disease. Majority of commercial serological tests use *F*. *hepatica* native antigens and indicate wide diversities in test accuracy. Nowadays, recombinant antigens have been introduced as diagnostic reagents offer better test standardization. A combination of highly pure recombinant antigens associated with correct folding will leads to improve specificity and sensitivity of ELISA for diagnosis of Fascioliasis. In this article, *Fasciola hepatica* saposin-like protein 2 (SAP-2), ferritin protein (Ftn-1) and leucine aminopeptidase (LAP) recombinant antigens were considered as tools for the detection of *F*. *hepatica* immunoglobulin G antibodies in persons with chronic human fasciolasis. The recombinant antigens were obtained as fusion proteins, expressed in *Escherichia coli* and purified by immobilized metal affinity chromatography (IMAC). The refolding processes of denatured recombinant proteins were performed using dialysis method in the presence of chemical additives, and reduced/oxidized glutathione (*in vitro*). The immunoreactivity of the recombinant antigens was assessed individually and in a combination compared with excretory/secretory antigen (E/S) in an enzyme-linked immunosorbent assay (ELISA) test. The experiments were optimized using 213 serum samples from humans, including patients with chronic fascioliasis, patients with other parasitic diseases, and healthy subjects. The results indicated 95% sensitivity and 98% specificity for rtFhSAP-2, 96% sensitivity and 91% specificity for E/S, 80% and 83.3% for rtFhFtn-1, 84% and 88% for FhLAP, and also, 96% and 95% for combination of recombinant antigens, respectively. In conclusion, the results of this investigation showed that rtFhSAP-2 with the highest specificity and acceptable sensitivity has a considerable superiority compared to mentioned antigens and even in combination with these antigens in serodiagnosis of human fascioliasis.

## Introduction

Fascioliasis is a food-borne zoonotic disease caused by liver trematodes of the genus *Fasciola*. The disease has been considered as a global disease of domestic ruminants and cause economic losses in livestock [1]. Nowadays, human fascioliasis has been recognized as an important reemerging parasitic disease by World Health Organization, with an estimated 17 million people affected worldwide [2]. *Fasciola hepatica* is the most wildly distributed species and predominates in temperate regions [3, 4]. Detection of *F*. *hepatica* infection by sensitive and specific methods is a key stage towards treating suspected patients. Clinically, the diagnosis of fasciolasis is difficult because the clinical symptoms are various and mimic those of a variety of other diseases. The conventional coprological diagnosis method is used as a gold standard for human fasciolosis. However, this method is not usually reliable and lacks sensitivity and reproducibility because eggs cannot recognize during the pre-patient period when liver damage has already happened by immature flukes. Additionally, eggs may not exist in the stool samples of infected patients with mature flukes since eggs are often released intermittently; also in the low parasitic burden of chronic cases, the eggs often be undetected [5]. Since anti-*Fasciola* antibodies can be detected after 2–7 weeks post-infection, serological tests such as Enzyme-linked immunosorbent assay (ELISA) can play a key role in the diagnosis of acute human fascioliasis when the eggs are not yet obvious in the stools [6–9]. Most of the available commercial serological kits, as well as in-house ELISAs, are based on excretory/secretory (E/S) products as antigen. This complex antigen has demonstrated high sensitivity for the diagnosis of infection, nevertheless, it often exhibits lower specificity due to the various level of cross reactivity between common antigens of *F*. *hepatica* in other helminths [10, 11]. In addition, lack of an efficient standard method for preparation of E/S antigen with a constant quality leading to inter-assay variability of serology tests [12–15]. One step forward for improving detection tests is to substitute the native antigens with recombinant proteins. In recent years, many studies showed that recombinant antigens may be an alternative source of antigens which are very useful for the serodiagnosis of fascioliasis. Using recombinant antigens would allow to standardize the tests better, reduce the expenses of production as well as more than one defined recombinant antigen can be used [16].

More recently, several recombinant proteins have been evaluated in an ELISA test to detect IgG antibodies [14, 15]. In this line, *F*. *hepatica* saposin-like protein 2 (FhSAP-2) has been suggested as one of the ideal antigens for development of ELISA kits that exhibited a highly strong antibody response in the acute and chronic phase of infection [14, 15, 17]. Moreover, the *F*. *hepatica* ferritin protein (FhFtn-1) protein has been shown a high reactivity with sera from experimental animals in all clinical phases of infection [18]. *F*. *hepatica* leucine aminopeptidase (FhLAP) is one of the most promising diagnostic markers that detected by sera of infected individuals with high accuracy [19–21].

There is solid evidence that *Escherichia coli* is the most common expression platform to produce recombinant proteins [22, 23]. However, a prevalent problem of *E*. *coli* is the absence of essential enzymes to create disulfide (S-S) bonds that leads to production of mis-folded proteins and Inclusion Bodies (IBs) [24]. Since the aforementioned proteins contain S-S bonds, refolding process is needed to recover protein folding and achieve higher antigenicity [25].

Consequently, for the first time, we presented very efficient methods to achieve *F*. *hepatica* recombinant antigens with the highest purity and proper folding in order to improve the antigenicity for diagnostic tests.

Despite the importance of using recombinant antigens in serological tests, there are few research studies about the combination of recombinant antigens in the diagnosis of fascioliasis [26].

Furthermore, a combination of recombinant antigens can be used as a promising diagnostic tool to enhance the sensitivity and specificity of ELISA tests. Thus in the present study, expression of rtFhSAP-2, rtFhFtn-1 and rFhLAP proteins were evaluated in several various conditions and different strains of *E*. *coli* so as to optimize the expression of protein. Furthermore, the diagnostic efficiency of the recombinant antigens was investigated, alone as well as in combination, in comparison with standard E/S antigen.

## Materials and methods

### Bioinformatics analysis

The hydrophobicity, secondary structure and S-S bonds of proteins were predicted by www.peptide2.com, SOPMA (Self Optimized Prediction Method with Alignment) (http://npsa-pbil.ibcp.fr/cgi-bin/) and http://clavius.bc.edu/~clotelab/DiANNA (DIANNA 1.1 web server).

### Serum samples

The number of 63 confirmed positive sera samples from individuals with chronic human fascioliasis, which were confirmed by the coprological method, was supported from medical diagnostic laboratories. Moreover, 78 serum samples from patients infected by other endemic parasites in Iran, which had been confirmed by a serological method, were collected to examine cross-reactivity reaction. The numbers of 72 negative control sera were gathered from healthy subjects from a non-endemic area of fascioliasis that had no evidence of *F*. *hepatica* eggs in their feces. All serum samples were collected as part of public health and clinical diagnostic activities, were available prior to the commencement of this study, Samples were obtained under informed written consent and provided by the Guilan University of Medical Sciences. This study was approved by the principals of the Ethics Research Committee of the Pasteur Institute of Iran.

## Bacterial strains, plasmids

The *E*. *coli* DH5α strain (Invitrogen, Carlsbad, CA, USA) was applied for maintenance and cloning. The *E*. *coli* Rosetta (DE3) (Promega, Madison, WI), SHuffle®T7 (New England Biolabs, Inc, USA) and BL21 (DE3) Origami were engaged for the expression of recombinant proteins. The pET28b, pET32b, and pET22b (Novagen, United Kingdom) plasmids were used for the construction of the expression system.

### Construction of the recombinant expression vector and sequence analysis

The DNA fragment of 270bp of FhSAP-2 gene, a fragment of 533bp of FhFtn-1 and a 1800bp DNA fragment from FhLAP were synthesized (without signal peptide regions) according to their sequence on GenBank database (Accession Nos. AF286903.1, HQ316639.1 and AY644459.1, respectively) in pGH plasmid by Generay Biotech co. (Shanghai, China). The tFhSAP-2, tFhFtn-1, and FhLAP DNA fragments were cleaved with *XhoI* and *NdeI* enzymes and then were ligated into pET28b, pET32b and pET22b, respectively in *XhoI*_*NdeI* sites. The presences of the fragment related to each gene in the recombinant vector were confirmed with sequencing by Macrogen co. (Seoul, Korea).

### Proteins expression

The Competent *E*. *coli* strains that mentioned above were transformed with pET28b-tSAP-2, or pET32b- tFhFtn-1 or pET22b-LAP and were grown in 250 ml Luria Bertani (LB) medium that was enriched with suitable antibiotics in terms of different strains and plasmids. Expression of recombinant proteins was incubated at OD600 from 0.3 to 0.6 under various concentrations of isopropyl-β-D-thiogalactopyranoside (IPTG) ranged from 0.1 to 8 mM, and also at various times (2, 4, 6 and 16 hr) to find the best expression conditions.

### Solubility analysis

Analysis of recombinant proteins solubility was carried out in *E*. *coli* strain as described previously [27]. One ml of each induced culture was pelleted and the crude cell extract was obtained in 10 ml of extraction buffer (0.1 mM EDTA, 1mM dithiothreitol (DTT), 30 mM Tris–HCl, pH 8 and then the mix was shaken in the cold room for 30 min. Suspension cells were disintegrated by sonication for 5 times (20s for each time). The pellet and the supernatant were then separated by centrifugation at 8000 rpm, 4˚C for 20 min.; and lastly, fractions were loaded onto SDS-PAGE.

### Washing of IBs and purification recombinant proteins under denaturing condition

rtFhSAP-2, rtFhFtn-1 and rFhLAP proteins were expressed under the optimum conditions in Rosetta strain. The pellet related to each protein were achieved from 250 ml culture media and suspended in 14 ml isolation buffer (350mM NaCl, 5 mM EDTA 50mM Tris HCl, 1 mM PMSF), the pH of buffer for rtFhSAP-2 and FhLAP proteins was 8 and for rtFhFtn-1was 7. The process of isolation as well as washing of IBs were performed the same as Mirzadeh et al [14]. The obtained pellet from washing of IBs, were suspended in 25 ml of binding buffer (Tris–HCl 30 mM, NaCl 500 mM, Urea 8 M and imidazole 2 mM, pH 8.0) (pH of binding buffer for purification of FhFtn-1 was 7), containing dithiothreitol (DTT) 10 mM and phenyl methyl sulfonyl fluoride (PMSF) 5mM. The cell suspensions were sonicated for 15 min and then centrifuged at 9000 rpm at 4°C for 30 min. The solubilized recombinant proteins were purified by affinity chromatography using a 5 ml column His-Trap™ FF (GE), which previously equilibrated with buffer binding. The column was washed with the binding buffer, containing 25 mM imidazole, and the proteins were then eluted with the elution buffer, containing 500 mM imidazole and 5 M urea at the flow rate of 2.0 ml/min. Finally, proteins concentration was determined by Bradford method using bovine serum albumin (BSA) as a standard.

### Refolding of recombinant proteins and western-blot analysis

The rtFHSAP-2, rtFhFtn-1 and rLAP solubilized IBs were first dialyzed in the renaturation buffer 1 (150mM NaCl, 1M urea,30 mM Tris-HCl, 0.1 mM EDTA, pH 8 (pH 7for refolding of FhFtn-1) for 24 hrs. at 50 rpm on incubating shaker (Taitec, BR-42FL, Japan) at 4°C. The dialysis bags then were transformed in the renaturation buffer 2 (50mM Tris–HCl, 100mM NaCl, 3mM reduced glutathione (GSH), 0.3mM oxidized glutathione (GSSG) and 0.2mM EDTA, 0.5 M urea, 10% glycerol, 0.5 mM arginine, pH 8.0) at 40 rpm for 48 hrs at 4°C on incubating shaker. Both dialyze methods were done in 100x dialysis buffer’s volume. The concentrations of recombinant antigens (His6-tSAP2, His6-tFhFtn-1, and His6-LAP) were determined by Bradford procedure. One μg of refolded proteins were evaluated by 12% SDS-PAGE, moved onto PVDF membrane and then probed with the sera samples of patients with chronic fascioliasis, the samples were loaded onto SDS-PAGE and lastly, separated proteins were moved onto polyvinyl difluoride (PVDF) (Millipore, Billerica, MA, USA). Membranes were blocked with 2% BSA in PBS containing 0.1% Tween 20 (PBS-T), pH 7.2 and probed with a pooled serum from patients with chronic fascioliasis (diluted 1/100) serum. The membranes were washed three times with PBS-T, and incubated with goat anti-human IgG antibody conjugated to horseradish peroxidase (HRP) (1:10000 dilution). Lastly, the protein bands were revealed using the peroxidase activity on 3, 3′-diaminobenzidine tetrahydrocholoride (DAB) substrate (Kem-En-Tec, Taastrup, Denmark) as the chromogenic substrate.

### The recombinant antigens for detection of *Fasciola* infection by ELISA

The ELISA test was performed as described previously [12], with a little modification. Briefly, Purified and refolded recombinant antigens were individually diluted in a range of concentrations (0.25–8 μg/ml) in carbonate/bicarbonate 0.05M (pH 9.6) in order to achieve optimized concentration, and incubated overnight at 4°C.

After examining the above condition, Maxisorb ELISA plates (Nunc, Roskilde, Denmark) were coated with the best concentration of rtFhSAP-2, rtFhFtn-1, rFhLAP and FhE/S antigens, in duplicate and placed overnight at 4°C. After three washes with PBS-T, the plates were blocked with 1% BSA in PBS-T at 37°C for 1 h. The serum samples were diluted 1/100 in blocking buffer and incubated at 37°C for 1 hr. The horseradish peroxidase-conjugated goat anti-human IgG antibody (Bethyl, Montgomery, TX USA) diluted 1/20,000 in the blocking buffer was added to the wells (100μl/well) and plates were incubated at 37°C for 45 min. After three times washing with PBS-T 3, 3′, 5, 5′- tetra methyl benzidine (TMB) (Kem-En-Tec) was added to each well. The plates then were incubated for 12 min in dark at room temperature. The reaction was stopped with the addition of 100 μl of 200 mM phosphoric acid. The optical density (OD) was measured at 450 nm by an ELISA reader (BioTek Instruments, Highland Park, VT) with a reference wavelength of 630 nm.

## Statistical analysis

Statistical analysis was carried out by the SPSS software ver. 22 (SPSS. Inc.). One-way ANOVA was used to compare the mean optical density (OD) reading values between the distinct groups, and *P* values < 0.05 were considered to show statistical significance. Moreover, area under the receiver operator characteristic (ROC) curves were calculated for each of recombinant proteins and E/S antigen using the GraphPad Prism software version 6.04 [28].

## Results

### Bioinformatics analysis

The results showed that rtFhSAP-2, rtFhFtn-1, and rFhLAP contain S-S bonds in their native structure and the most part of proteins is Hydrophobic (Table 1).

**Table 1:** Bioinformatics analysis of target proteins.

**Protein**	**Hydrophobic/Hydrophilicity Analysis**				**Secondary structure**				**Disulfide bond**
	Hydrophobic	Acidic	Basic	Neutral	Alpha helix	Extended strand	Beta turn	Random coil	
FhSAP2	46.53	14.85	10.89	27.72	67.33	11.88	3.96	16.83	31–62 34–101 71–95
FhFtn-1	37.34	8.86	16.46	37.34	48.73	12.03	10.76	28.48	59–66 95–146
FhLAP	45.53	11.09	13.77	29.45	41.49	17.97	8.60	31.93	30–101 90–294 364–449

Analysis of the hydrophobicity, secondary structure and disulfide bonds of three *F*. *hepatica* proteins in order to identifying their native conformation

### Cloning of rtFhSAP-2, rtFhFtn-1 and rFhLAP

The DNA fragments encoding tFhSAP-2, tFhFtn-1, and FhLAP amino acids were correctly cloned in expression plasmids. Sequence analysis of the inserted tFhSAP-2, tFhFtn-1 and FhLAP displayed 100% identity with the sequence published of *F*. *hepatica* in GenBank (AF286903.1, HQ316639.1, and AY644459.1) (data not shown). The plasmids pET28b, pET32b, and pET22b were used for the construction of the (rtFhSAP-2, rtFhFtn-1 and rFhLAP) expression system, respectively.

### Optimization of proteins expression and purification

**Fig 1:**
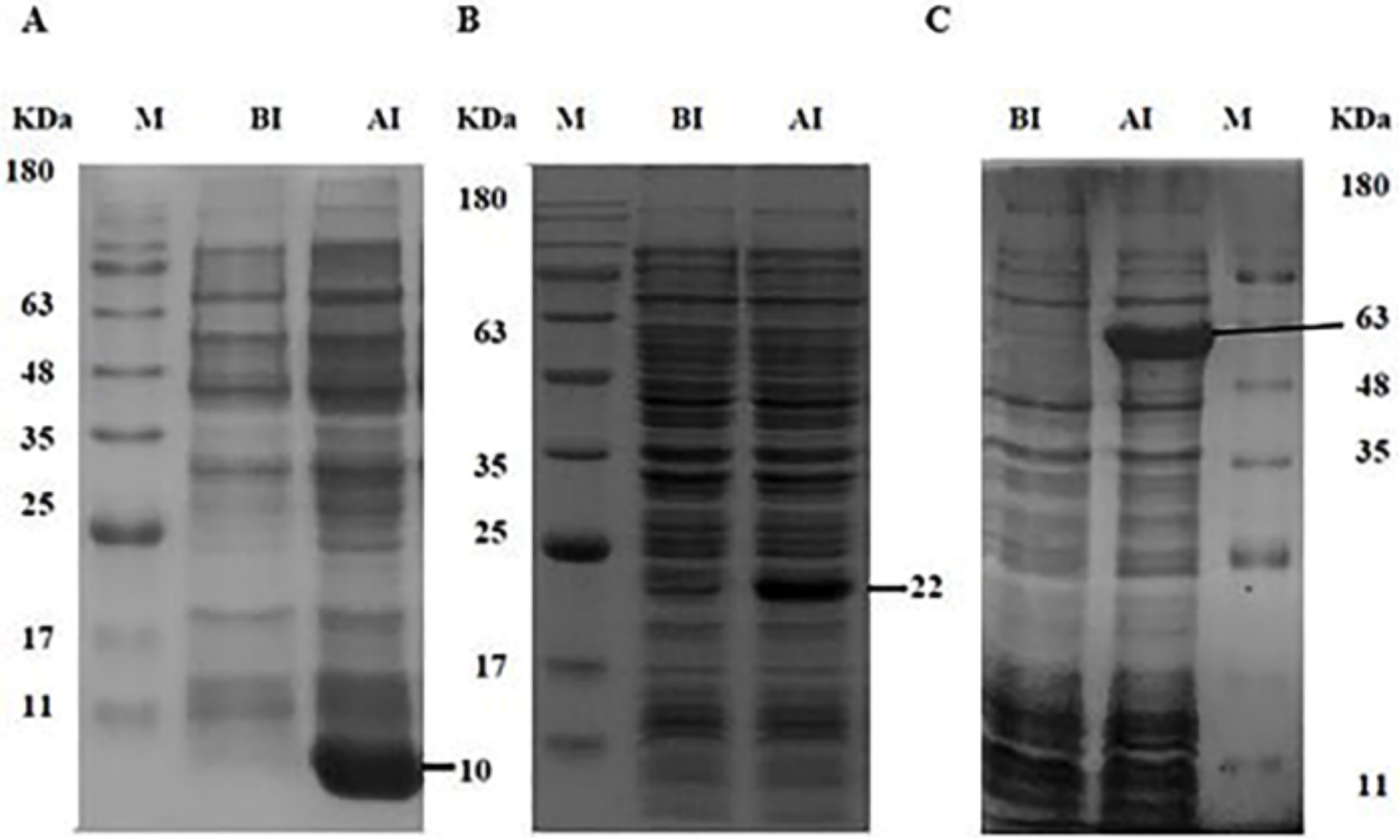
Optimization of recombinant proteins expression in Rosetta (DE3) strain. The expression of (A) rtFhSAP-2 (B) rtFhFtn-1 and (C) rFhLAP. Bacterial samples which were taken just before (BI) and after induction (AI) with IPTG, examined on 12% SDS-PAGE.

**Fig 2:**
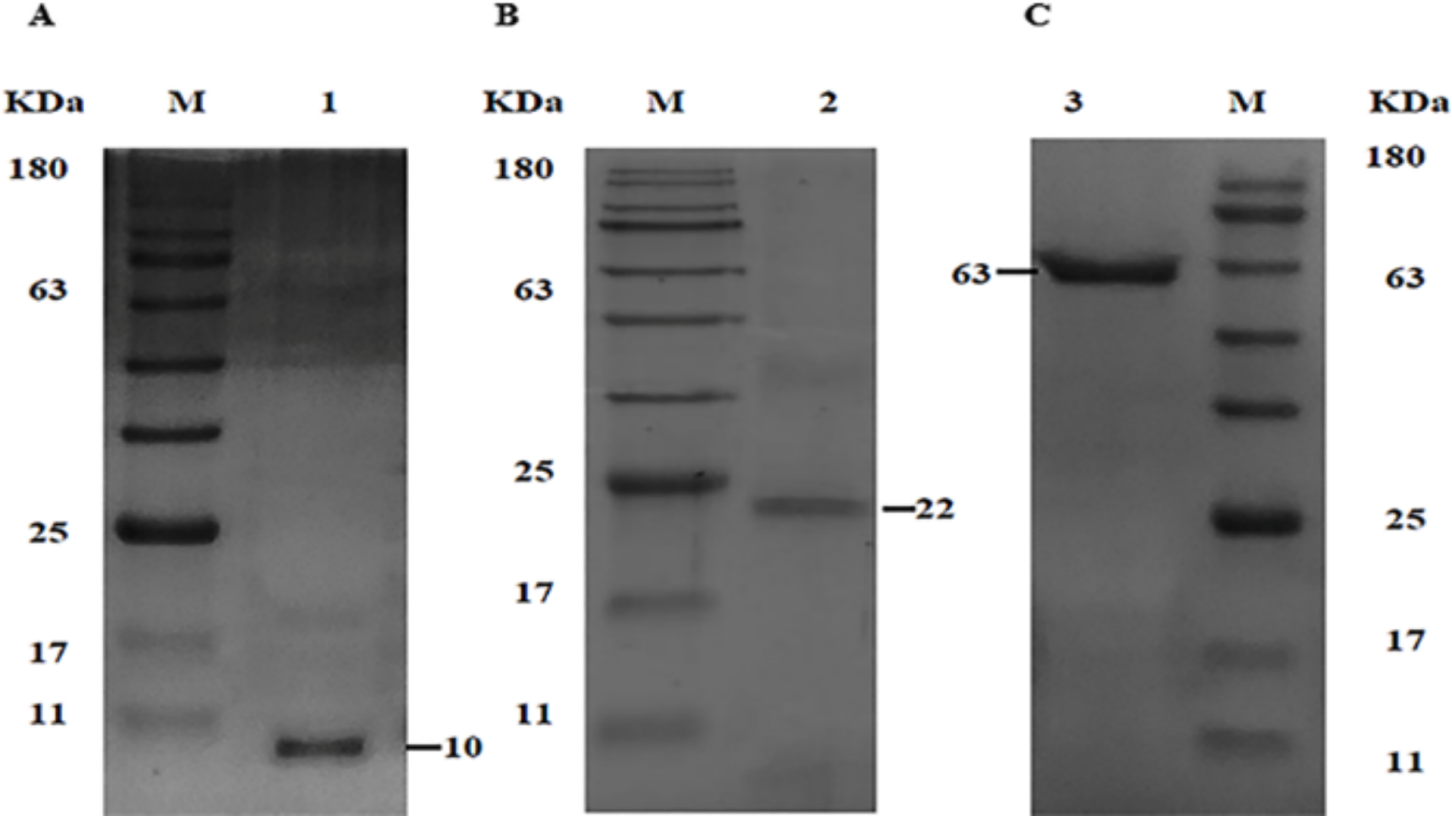
Purification of soluble form of rtFhSAP-2, rtFhFtn-1 and rFhLAP proteins. (A) Purified (eluted) rtFhSAP-2 after IMAC (1). (B) Purified (eluted) rtFhFtn-1 after IMAC (2). (C) Purified (eluted) rFhLAP after IMAC (3).

The recombinant plasmids pET28b-tFhSAP-2, pET32b-tFhFtn-1, and pET22b-FhLAP were transformed into *E*. *coli* (DE3) strains. In order to optimize the expression of recombinant proteins, we studied the effect of diverse conditions. The optimum expression was achieved in Rosetta (DE3) strain at IPTG concentration of 0.2mM, cell density (OD600) of 0.6, induction time of 3 hrs, with temperatures 30°C. SDS-PAGE analysis of the induced bacteria showed protein bands of rtFhSAP-2, rtFhFtn-1 and rFhLAP corresponding to their real size which were approximately 10, 22 and 63 KDa respectively (Fig 1). In our study, most of the defined proteins were found in insoluble form in Rosetta (DE3) strain (data not shown). Therefore, isolation and lysis buffers containing urea and Triton X-100 along with centrifugation were applied to separate their IBs from *E*. *coli* impurities. The solubilized IBs were purified by IMAC method and eluted from the column as a single sharp peak by the buffer containing 2M urea and 500 mM imidazole. In addition, the result of the SDS-PAGE analysis showed that the eluted proteins had purity higher than 95% for each of protein (Fig 2).

### Antigenicity evaluation of refolded proteins

**Fig 3:**
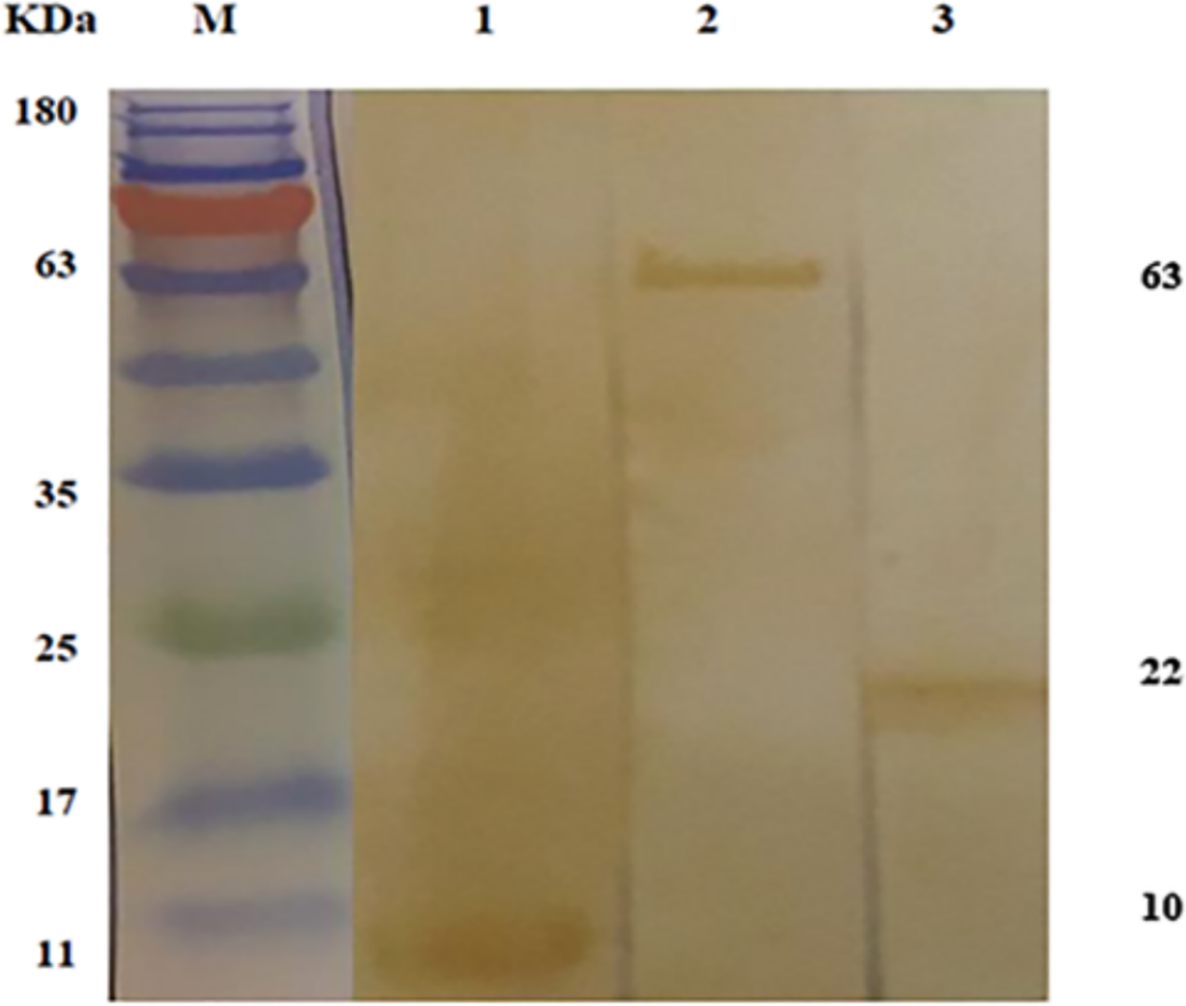
Western blot analysis of refolded proteins. Reactivity of rtFhSAP-2 (1), rFhLAP (2) and rtFhFtn-1(3) proteins against serum samples of infected patients with *Fasciola*.

Immunogenicity of refolded rtFhSAP-2, rFhLAP, rtFhFtn-1 proteins was confirmed by the western-blotting method. One μg of refolded proteins was analyzed by 12% SDS-PAGE, transferred onto PVDF membrane and then probed with the serum samples of patients with chronic fascioliasis. The results showed specific bands approximately 10, 63 and 22 KDa respectively, with accordance to their real size that were highly antigenic with Fascioliasis patients' samples (Fig 3).

### Immunological detection of refolded proteins by ELISA

**Fig 4:**
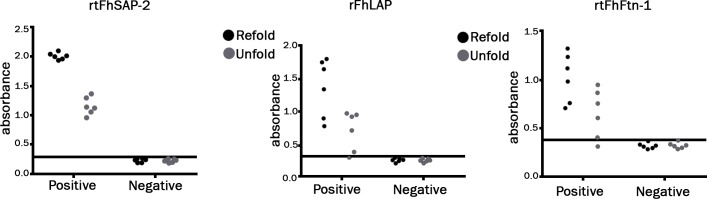
Analysis of the reactivity folded recombinant proteins with their unfold forms. Serum samples obtained from subjects with fascioliasis. Each recombinant proteins sample was assessed in triplicate.

Evaluation of ELISA for rtFhSAP-2, rtFhFtn-1, rFhLAP proteins as well as E/S were carried out at the best concentration of rtFhSAP-2(0.25 μg/ml), rtFhFtn-1(2 μg/ml), rFhLAP(1 μg/ml) and E/S (10 μg/ml) antigens, mix of recombinant antigens with identified concentrations were coated in duplicate as well (Fig 4 and Table 2).

**Table 2:** Comparison the reactivity of folded rtFhSAP-2, rtFhFtn-1and rFhLAP with their unfolded forms by ELISA.

Concentrations of proteins (μg/ml)	rtFhSAP2		rtFhFtn-1		rFhLAP	
	Refold	Unfold	Refold	Unfold	Refold	Unfold
8	2.006	1.128	1.243	0.948	1.788	0.975
4	2.102	1.139	1.127	0.873	1.747	0.954
2	2.042	1.299	1.329	0.761	1.645	0.931
1	1.938	1.056	0.988	0.611	1.332	0.719
0.5	1.961	0.964	0.712	0.401	0.890	0.389
0.25	2.016	1.371	0.764	0.317	0.791	0.362

Recombinant proteins samples were assessed in triplicate in defined concentrations.

The difference between negative samples (refold and unfold forms) were narrow.

**Fig 5:**
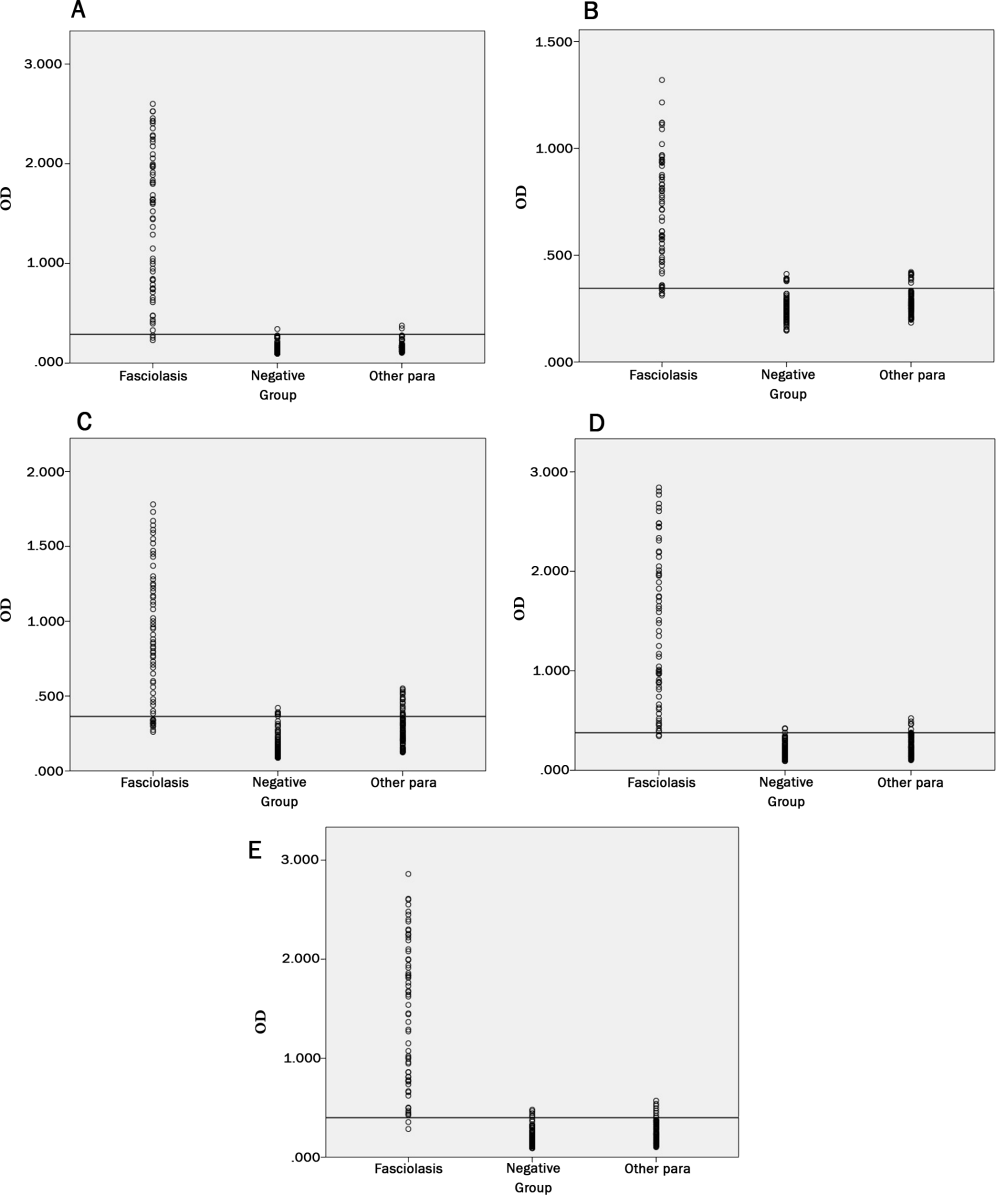
ELISA assay showing the reactivity of rtFhSAP-2, rtFhFtn-1, rFhLAP, and combination. (A) Scatter of rtFhSAP-2 ELISA absorbance value. (B) Scatter of rFhLAP ELISA absorbance value. (C) Scatter of rtFhFtn-1 ELISA absorbance value. (D) Scatter of combination ELISA absorbance value. (E) Scatter of E/S ELISA absorbance value, Horizontal line indicates the cutoff value established in both assays.

The results of ELISA showed that reactivity of rtFhSAP-2 against *F*. *hepatica* antibody is highly more than other antigens (Fig 5 and Table 3). In the rtFhSAP2-ELISA assay, the OD450 values for the negative serum samples ranged between 0.096 and 0.345. The cutoff point led to 0.289 (line in Fig 5 and Table 3). Sera from patients with fascioliasis showed a range between 0.23 and 2.526. The area under the ROC curve for E/S, rtFhSAP-2, combination of antigens, rtFhFtn-1 and rFhLAP were 0.988, 0.996, 0.992, 0.945 and 0.979, respectively

**Table 3:** Statistical analysis of the results obtained with rtFhSAP2, rtFhFtn-1, rFhLAP and a combination of them, beside E/S antigen in ELISA; all of the positive serum were confirmed by the gold standard.

Antigens	E/S			rtFhSAP2			rtFhFtn-1			rFhLAP			Recombinant antigens Combination		
ELISA	P	N&C	Total	P	N&C	Total	P	N&C	Total	P	N&C	Total	P	N&C	Total
Positive (Number of serum sample)	61	13	74	60	3	63	51	25	76	53	18	71	61	7	68
N&C (Negative and Cross) (Number of serum sample)	2	142	144	3	152	155	12	130	142	10	137	147	2	148	150
Total	63	155	218	63	155	218	63	155	218	63	155	218	63	155	218
Cut-off	0.395			0.298			0.364			0.345			0.378		
Sensitivity (SE)	96%			95%			80%			84%			96%		
Specificity (SP)	91%			98%			83.3%			88%			95%		
Accuracy	93%			97%			83%			87%			95%		
p-Value	0.05			0.05			0.05			0.05			0.05		

P (positive), N&C (Negative and Cross), SE (sensitivity), SP (specificity).

## Discussion

Gold standard for the diagnosis of fasciolosis is the conventional coprological method. Nevertheless this approach is not reliable inthe acute phase of the disease and has poor sensitivity during the chronic phase [5]; therefore antibody detection is one of the most convenient tests, allowing diagnosis in the early stage of human fascioliasis compared to the coprological test [6]. Diagnosis of IgG antibodies in human serum by ELISA is considered as a sensitive diagnostic tool to identify acute and chronic phase [29]. Due to the high complexity of antigenic structure among helminthes, a variable level of cross reactivity could be elicited; consequently, developing serological test with high specificity is a priority [26].

E/S product is one of the most common antigens which use in the diagnosis of fascioliasis. Although the use of this complex antigen has been introduced as a sensitive tool for early detection of infection, the cross reactivity decreases the specificity and makes antigen’s standardization difficult in diagnosis [6, 11, 26]. In contrast, a recombinant protein exhibits a constant composition leading to a reduction of cross reactivity and higher accuracy in ELISA test [30]. Recently, it has been demonstrated that the development of proper mixtures of recombinant proteins improves the test performance in the diagnosis of infections. Furthermore, it has been shown that obtaining large quantities of highly pure recombinant *F*. *hepatica* antigens with correct folding play a vital role in improvement of antigenicity and accuracy in serodiagnosis methods [14, 27]. In this line, we evaluated the immunodiagnostic potential of tFhSAP-2, tFh-Ftn-1 and FhLAP recombinant antigens, alone or in combination, for the detection of IgG antibodies in fascioliasis by ELISA. These antigens were selected as a result of high diagnostic value with reasonable sensitivities and desirable specificity when assessed individually against a limited sample of homologous and heterologous sera [13, 17, 19, 30–32]. Analysis of different cloning and variations of purification methods have shown diverse levels of sensitivity, specificity, and accuracy in the diagnostic tests. One of the most important reasons of diversity is that recombinant proteins produced in *E*. *coli* are highly likely to lose their antigenic value because of incorrect folding [33]. Therefore, first of all, the structural aspects of three *F*. *hepatica* antigens were predicted by bioinformatic methods. The results showed that rtFhSAP-2, FhLAP and rtFhFtn-1 have S-S bonds in their native structure and the highest percentage of recombinant proteins are hydrophobic (Table1); consequently, the most amounts of the target proteins cannot fold into their native conformation and instead accumulated in IBs. Thus, recovering soluble and correctly folded proteins from unfolded IBs not only require washing, IBs isolation, solubilization and purification processes but also need a refolding method to achieve their native 3D structure and conformational epitopes [33–35].

Accordingly, we expressed recombiant proteins under different conditions along with the diverse expression vectors and strains to obtain optimized recombinant proteins, (Fig 1). After proteins isolation with the highest purity (Fig 2), refolding process was performed using dialysis process in the presence of chemical additives, and reduced/oxidized glutathione (*in vitro*), which is known as a highly efficient method [14, 36]. The results proved that refolding proteins are highly more efficient in diagnosis of IgG antibody compared to unfolded proteins (Table 2 and Fig 4).

In addition, ELISA was carried out with *E*. *coli* antigen as a control, expressed and purified in the same way as the *F*. *hepatica* recombinant antigens [27]. The folded recombinant proteins alone and in combination was compared to E/S antigen in ELISA test, it was illustrated that the sensitivity of antibody diagnosis obtained from the combined antigens was the same as E/S antigen at 96% and slightly more than rtFhSAP-2 (95%). Whereas the sensitivity of rFhLAP and rtFhFtn-1 were substantially lower than both of the combination set and rtFhSAP-2 at 84% and 80%, respectively (Table 3 and Fig 5). Moreover, the ELISA survey of E/S and combined recombinant antigens showed the highest absorbance of positive OD readings reaching 2.86 and 2.52 respectively, and false-negative reactions were observed in only three positive sera. In contrast, the lowest absorbance reaching 0.713 was gained with rFhLAP. RtFhFtn-1 had the highest number of false positive at about 11 serums, whereas rtFhSAP2 was more specific in relation to the sera of both healthy people and patients infected with other parasites. The specificity of a combination of three antigens and E/S was lower, at 95% and 91%, respectively than that of rtFhSAP-2 at 98%. Furthermore, the accuracy of a combination set, rFhLAP and rtFhFtn-1 were lower than that of rtFhSAP-2. In agreement with our findings, a study by Gottstein et al. revealed that using the mixture of two *F*. *hepatica* recombinant proteins, rtFhSAP-2 and cathepsin L-1 (recCL1), revealed the lower accuracy results in comparison with rtFhSAP-2 antigen alone [26]. It should be noted that although our results revealed the similar sensitivity of 96% for both of combination antigens and E/S, the acceptable sensitivity was also obtained for rtFhSAP-2 (95%). On the other hand, the highest specificity and accuracy was determined for rtFhSAP-2 compared to other antigens, suggesting rtFhSAP-2 as a reliable marker for detecting of fascioliasis, more favorable than both of combination and E/S. A number of studies demonstrated that the mixtures of several recombinant antigens which offer multiple different epitopes are more likely to detect *F*. *hepatica* IgG antibodies during different stages of the infection. Given that the antigen combination could decrease the specificity due to the common epitopes shared with other antigens present in helminthes [10, 37]; this issue could be suggested as a reason for a slight reduction in sensitivity of a combination set. In the current study, the lowest specificity was obtained for rtFhFtn-1; hence, it seems that this protein might have more common epitopes with proteins of other parasites compared to other antigens in this essay.

Overall, although the results of refolded rtFhSAP-2 in this article compared to a mixture of recombinant proteins are promising, the further survey is needed to identify desirable candidates and mix with refolded rtFhSAP-2. In addition, the discovery of new infection antigens and improvement of the efficiency of purification and refolding process in order to increase diagnostic accuracy by recombinant antigens are needed, particularly those which have high specificity. Moreover, analysis of folding kinetics for evaluation of refolded proteins by circular dichroism is required to obtain more knowledge regarding the protein configuration.
